# 

**Published:** 1872-09

**Authors:** 


### Contents of September Number.

### ORIGINAL DEPARTMENT.

ART. I.—The Thermometer in Disease. By F. M. Hamlin, M. D...... 41
ART. II.—Stricture of the Esophagus. By James S. Bailey, M. D...... 49
ART. III.—Veratrum Viride in Pulmonary Hemorrhage. By H. N. Eastman, M. D. ........................................ 51

### TRANSLATIONS.

On the Recent advances in the Theory of Vision. By H. Helmholtz. Translated by F. W. Abbott, M. D.,.......................... 57

### MISCELLANEOUS.

The Proper Definition of the Word Cure, as applied to Medicine. By Paul F. Eve, M. D.......................................... 66
The Synovial Membranes in Pyæmia. By Robert Hamilton, F. R. C. S.. 69
Items and Selections. By E. N. Brush.............................. 71

### EDITORIAL DEPARTMENT.

Inferior and Impure Drugs.......................................... 73
Buffalo State Asylum for the Insane. Laying of the Corner Stone....... 74
Sad Bereavement.................................................. 75
Commencement of the College Course .............................. 75
Books Reviewed:
A System of Surgery. By Samuel D. Gross, M. D., LL. D: D. C. L. Oxon........................................ 75
The Correct Principles of Treatment for Angular Curvature of the Spine. By Benjamin Lee, A. M., M. D......... 76
Lithotomy and Lithotrity. By Gordon Buck, M. D. ....... 77
Diseases of the Throat. By J. Solis Cohen, M. D.......... 78
A Manual of Qualitative Analysis. By Robert Galloway, F. C. S ................................................ 78
Archives of Ophthalmology and Otology. Vol. II. No. II.. 79
Books and Pamphlets Received,........................................ 79

